# Diagnosis of Early Mycosis Fungoides

**DOI:** 10.3390/diagnostics11091721

**Published:** 2021-09-19

**Authors:** Tomomitsu Miyagaki

**Affiliations:** Department of Dermatology, St. Marianna University School of Medicine, 2-16-1 Sugao, Miyamae-ku, Kawasaki 216-8511, Kanagawa, Japan; asahikari1979@gmail.com; Tel.: +81-44-977-8111; Fax: +81-44-977-3540

**Keywords:** mycosis fungoides, early stage, diagnostic algorithm, T-cell receptor rearrangement, tumor-specific marker, microRNA

## Abstract

Mycosis fungoides (MF), the most common type of cutaneous T-cell lymphomas, generally has a favorable clinical course. Early MF typically presents erythematous patches and/or plaques and lasts for many years without affecting the life expectancy. Only limited cases progress to develop skin tumors, with subsequent lymph nodes and rarely visceral organ involvement. One of the clinical problems in early MF is the difficulty in differentiating the disease from benign inflammatory disorders (BIDs), such as atopic dermatitis, chronic eczema, and psoriasis. In some MF cases, clinical and pathological findings are similar to those of BIDs. However, the accurate diagnosis of early MF is quite important, as inappropriate treatment including immunosuppressants can cause unfavorable or even fatal outcomes. This article focuses on general methods and novel tools for diagnosis of early MF.

## 1. Introduction

Mycosis fungoides (MF) is the most common type of cutaneous T-cell lymphomas (CTCLs), a heterogenous group of non-Hodgkin lymphoma of T-cell origin that is defined to primarily present in the skin, representing almost 50% of all CTCL cases [1,2]. MF is characterized by malignant proliferation of CD4^+^ T cells with epidermotropism in the skin and generally has a prolonged clinical course. In early stages, the disease typically presents in the form of erythematous patches and/or plaques and this stage can last for many years without clinical progression and affecting the life expectancy of patients [1,3,4,5,6]. A part, but not all, of such patients progress to develop skin tumors, with subsequent lymph node and rarely visceral organ involvement and they are regarded as having advanced-stage disease [1,3,4,5,6]. Guidelines describing the diagnosis of MF are created by various professional societies [2,7,8,9], and the methods for diagnosis are mostly consistent in those guidelines. Generally, the diagnosis of MF is made comprehensively based on clinical presentation, clinical course, pathological and immunohistochemical analysis, and occasionally molecular biological analysis. Nevertheless, the diagnosis of MF, especially early MF, is still challenging. It is sometimes hard to differentiate early MF from benign inflammatory disorders (BIDs), such as atopic dermatitis (AD), chronic eczema, and psoriasis [10,11,12], because in some MF cases, clinical and pathological findings are similar to those of BIDs. In addition, the difficulty in differential diagnosis can also be caused by the lack of tumor cell-specific markers and not enough sensitivity and specificity of genetic tests detecting clonality of tumor cells. The accurate diagnosis of early MF is quite important in selecting therapeutic strategy. There have been many MF cases that follow an unfavorable or even fatal outcome due to inappropriate treatment including immunosuppressants and dupilumab based on the misdiagnosis as BIDs [10,11,13]. Here, I summarize the general features, algorithm for diagnosis, and novel suggested diagnostic tools of early MF.

## 2. General Features of Early MF for Diagnosis

**Figure 1:**
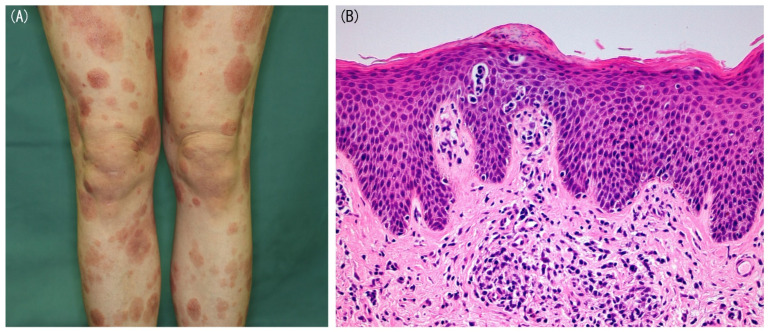
(**A**) Clinical presentation of classical early mycosis funogides (MF). Well-demarcated erythematous patches and plaques with occasionally poikiloderma are shown. (**B**) Pathological findings of early MF (hematoxylin-eosin, original magnification ×100). Epidermotropism of atypical lymphoid cells is shown.

Clinical presentation is one of significant factors in the diagnosis of early MF, although similar findings can be seen in some BID cases. The presence of a rare MF variant with a single lesion, unilesional MF, is well-known [14], whereas most MF cases show multiple lesions. Early MF typically presents well-demarcated erythematous patches and/or plaques with occasionally poikiloderma and the lesions are characterized by variability in the size, shape, and color (Figure 1A). Initially, MF lesions have predilection to non-sun-exposed areas, such as buttock, flanks, inner thighs, and inner arms. However, in folliculotropic MF, the most common variant of MF, lesions may appear on the face or scalp early in the clinical course [15]. Clinical course can also help the diagnosis of MF and the most important feature is the persistent nature of the disease. MF lesions tend to increase in size and number over time without treatment or even under the treatment with topical corticosteroids. Complete response rates by class I topical steroid were reported to be 63% and 25% in early MF patients with T1 stage (less than 10% of skin involved) and T2 stage (10% or more of skin involved), respectively [16]. Therefore, in many MF patients, topical steroids fail to clear the lesions completely. Moreover, in cases with complete remission, the lesions usually recur when the treatment is stopped or newly develop in the untreated areas.

Pathological analysis of the lesional skin is mandatory in the diagnosis of early MF. The pathological features in early MF are as follows: (1) the presence of atypical lymphoid cells with slight larger size than normal lymphocytes and cerebriform, hyperchromatic nuclei; (2) the distribution of lymphocytes singly or in small collections in an epidermis devoid of spongiosis, also called disproportionate epidermotropism; (3) individual haloed atypical lymphocytes within the epidermis; (4) alignment of single atypical lymphocytes along the dermal-epidermal junction; (5) fibrosis of the papillary dermis and (6) a band-like infiltrate in the dermis [12,17]. The presence of atypical lymphoid cells in the epidermis may be the most important pathological feature of early MF (Figure 1B), whereas in some MF cases, cell or nuclear atypia and epidermotropism are not remarkable. Epidermotropism-like findings or mild atypia of infiltrating lymphocytes can also be seen in BIDs. Collectively, differentiating early MF from BIDs based on pathological findings is quite difficult in some cases. Other than above findings, Dalton et al. reported that eosinophil infiltration with more than three cells per tissue section was rarely found in early MF, suggesting that eosinophil infiltration extent in lesional skin may be useful in the differential diagnosis between early MF and BIDs [18]. Anyway, repeated biopsies or multiple biopsies from various lesions may be needed for the accurate diagnosis for early MF. To enhance the pathological characteristics, topical treatment should be discontinued 2 to 4 weeks before skin biopsy.

**Figure 2:**
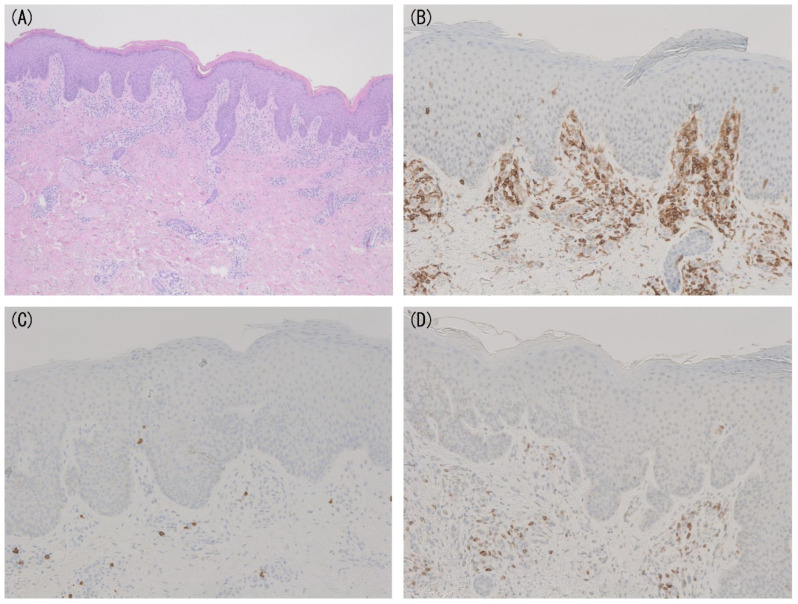
(**A**) Pathological findings of mycosis fungoides (MF) without epidermotropism (hematoxylin-eosin, original magnification ×40). (**B**–**D**) Immunohistochemical findings of CD4 (**B**), CD8 (**C**), and CD7 (**D**) in the case shown in (**A**) (original magnification ×100). The elevation of CD4/CD8 ratio and loss of CD7 are shown.

Immunohistochemical analysis of some surface molecules may also contribute to the diagnosis of MF. The tumor cells of MF are usually positive for CD3 and CD4 and negative for CD8 [1]. The elevation of CD4/CD8 ratio greater than 4–6 may suggest the proliferation of neoplastic CD4^+^ T cells and the diagnosis of MF (Figure 2A–C) [19]. However, it should be taken into consideration that Langerhans cells and histiocytes are also positive for CD4. The loss of pan T-cell markers, such as CD2, CD5, and CD7, in CD4^+^ T cells in lesional skin also supports the diagnosis of MF. Among them, the loss of CD2 and CD5 is rarely found in early MF. CD2 or CD5 expression by less than 50% of infiltrating T cells is completely specific but only about 10% sensitive for MF [17]. On the other hand, diminished CD7 expression is more frequently seen in early MF (Figure 2D), whereas it can also be shown in some BID cases [17,20]. Extremely decreased CD7 expression (less than 10% infiltrating lymphocytes) was reported to be 41–80% sensitive and 93–100% specific for the diagnosis of MF [20,21]. As the number of tumor cells in the dermis is limited in early MF, the lack of such T-cell markers may be seen in only epidermis in some cases.

The detection of monoclonality of T-cell receptor (TCR) gene by polymerase chain reaction (PCR) or Southern blot analysis is also an important finding in MF and can be a diagnostic clue in the cases that mimic BIDs both clinically and pathologically. PCR analysis is more sensitive than Southern blot analysis [22]. Southern blot analysis could fail to detect monoclonality in many early MF cases [23] and thus, PCR analysis is more frequently used in the diagnosis of early MF. The recent report showed that clonal TCR gene rearrangement was demonstrated in 83% of early MF cases by PCR analysis [24]. However, due to high sensitivity, the presence of monoclonality by PCR can be seen in some BID cases, because not monoclonal but oligoclonal accumulation of T cells occurs in BIDs [25,26,27]. Detection of identical clones from two different sites was reported to be highly specific for MF [28].

## 3. Algorithm for Diagnosis of Early Mycosis Fungoides

The diagnosis of early MF is made comprehensively based on combined findings described above. In 2005, the International Society for Cutaneous Lymphoma proposed the algorithm for diagnosis of early classical MF (Table 1) [17]. When a sum total of four or more points is achieved, the diagnosis of MF is made. Compared to immunohistochemical and molecular findings, clinical and pathological findings are regarded as more important. If the patient meets the basic and two or more additional criteria of clinical and pathological findings, the diagnosis of early MF can be made without immunohistochemical and molecular analyses. On the other hand, even if the patient meets the immunohistochemical and molecular criteria, additional clinical and/or pathological findings are needed.

The validity of the algorithm was first evaluated by Vandergriff et al. in 2015 [29]. They retrospectively applied the algorithm to 24 early MF patients and 10 patients with skin diseases mimicking MF, such as eczema, drug eruption, and psoriasis. Twenty-one out of 24 early MF patients met or exceeded the four-point threshold, while four points were achieved only in four MF mimics, and none achieved five or six points. The sensitivity and specificity were 87.5% and 60% respectively and the algorithm was found to be a statistically valid for distinguishing MF from its mimics. As the analysis of TCR clonality is unavailable in some facilities and detection rates depend on the methods, some group assessed the validity of the algorithm excluding the molecular biological criteria. Amorim et al. retrospectively reviewed 67 early MF patients clinically, pathologically, and immunohistochemically [30]. They found that 43 of 67 patients (64%) met the basic and two or more additional criteria of clinical and pathological findings and the diagnosis of early MF could be made by those findings. Moreover, when immunohistochemical analysis was added, 61 of 67 patients (91%) met the criteria for the diagnosis of early MF. Similarly, the other group also showed that the sensitivity of the algorithm excluding biological molecular criteria was 93%, while the algorithm including the criteria achieved 100% sensitivity [31]. Collectively, the algorithm is highly sensitive and most early MF cases can be diagnosed accurately, but the specificity has not yet been validated sufficiently. The modification of the algorithm to improve the specificity and sensitivity may be desirable.

## 4. Novel Diagnostic Markers of Early MF

The difficulty in differential diagnosis between early MF and BIDs may be partially caused by the lack of tumor cell-specific markers. Thymocyte selection-associated high mobility group box factor (TOX), belonging to DNA-binding factors, has the capacity to regulate the double dull to CD4^+^CD8^low^ transition during positive selection of T cells [32]. After positive selection, TOX expression disappears from CD4^+^ T cells before they exit the thymus [32]. Early studies reported TOX to be a tumor cell-specific marker of CTCLs including early MF based on immunohistochemical findings that TOX was expressed in tumor cells of CTCLs but hardly in inflammatory infiltrates of BIDs [33,34]. However, more recent reports found that TOX was also expressed in infiltrating lymphocytes in BIDs, although the frequency was not high [35,36,37]. Positive TOX expression was identified in 74% of MF cases and in 32% of BID cases and normal skin [37]. Other group reported that TOX was expressed by more than 50% of tumor cells in 83% of MF cases, whereas only 2% of inflammatory dermatoses cases showed TOX expression in more than 50% infiltrating lymphocytes [36]. More recently, the report from Egypt revealed that TOX can be a potential diagnostic marker differentiating hypopigmented MF from early active vitiligo [38]. TOX expression was found in 93% of hypopigmented MF, while only 7% of vitiligo was weakly positive for TOX. Unfortunately, TOX is not considered as a tumor cell-specific marker, but TOX expression can be an adjunctive diagnostic marker, similar to loss of pan T-cell markers, and might be added in the diagnostic algorithm for early MF.

Cell adhesion molecule 1 (CADM1), one of adhesion molecules, is a well-known tumor suppressor gene in a variety of human cancers [39]. On the other hand, interestingly, CADM1 is overexpressed in tumor cells of adult T-cell leukemia/lymphoma (ATLL) and involved in oncogenesis [40]. As CADM1 is not expressed on normal T cells, it can be a diagnostic marker for ATLL [41]. Recently, CADM1 was reported to be a potential diagnostic marker also in MF. Yuki et al. revealed that 55 of 58 MF cases including 34 early cases showed CADM1 expression in more than 5% of infiltrating lymphocytes, while CADM1 expression was found in less than 5% of infiltrating lymphocytes in all 50 BID cases [42]. Although further validation from other groups is required, CADM1 can be a potential diagnostic marker for early MF.

## 5. Next-Generation High-Throughput Sequencing

The assessment of TCR clonality by PCR relies on length determination of the most abundant PCR product assumed to represent the predominant TCR clone. TCR clonality by PCR can be detected in a small number of BID patients, while some early MF patients who have limited number of malignant cells do not present the clonality as described above. This lack in the test’s sensitivity and specificity for the detection of clonality of tumor cells also makes it difficult to differentiate early MF from BIDs. Recently, the application of next-generation high-throughput sequencing (NGS) to the detection of malignant clones in CTCL has been introduced by multiple groups. By sequencing the third complementarity determining regions (CDR3) of TCRβ and TCRγ genes, the total amount and frequencies of the individual T-cell clones can be quantified and the unique nucleotide sequences of each clone’s CDR3 regions can be detected [43,44]. Based on the presence of a dominant CDR3 sequence, malignant proliferation of the clone can be identified. Dominant malignant clones were detected in 100% of MF and SS patients without the frequency criteria [45,46]. When the cases with the most frequent two TCR sequences were accounted for, over 5% of the total reads were regarded as clonal and 85% of the MF cases showed clonality [44]. The sensitivity of the NGS method is superior to that of the PCR method. On the other hand, due to its high sensitivity, expanded T cell clones were also detected in BIDs, similar to the PCR method. Although the specificity of the NGS method was also reported to be better than the PCR method [47], Kirsch et al. showed that the top clone frequency with respect to the remaining T cell population without the threshold criteria failed to distinguish CTCL from BIDs [45]. They suggested using the absolute number of clonal T cells in a particular unit of skin evaluated by the frequency of top T cell clone among total nucleated cells as a distinguishing parameter. The parameter was reported to discriminate CTCL clearly from BIDs. However, calculating this parameter is very complicated and more easier criteria may be required. Quite recently, Zimmermann et al. sought to define the optimal criteria for T-cell clonality by NGS using 101 CTCL samples including 47 early MF samples and 43 BID samples [48]. With 5% and 25% top clone frequency thresholds, the specificities for CTCL diagnosis were 95% and 100%, and sensitivity 89% and 50%, respectively. They concluded that 5% top clone frequency threshold may be useful for diagnosis of CTCL including early MF. It will take a long time to generalize NGS in multiple clinical facilities, but NGS can be an important tool in the diagnosis of early MF in the future.

## 6. MicroRNA for the Diagnosis of Early MF

MicroRNA (miR) profiles have been widely studied in CTCL and dysregulated expression of various miRs have been reported [49]. Given that miR profiles are varied and unique depending on the diseases including BIDs and various cancers, aberrant miR expression in CTCL may contribute to the differential diagnosis from BIDs. The potential differential diagnostic utility of miR profiles between CTCL and BIDs was first reported in 2011 [50]. Ralfkier et al. found that miR-326, miR-663b, and miR-711 were highly induced in CTCL and that miR-203 and miR-205 were repressed by microarrays. The expression levels of these five miRs could distinguish CTCL from BIDs with >90% accuracy. As microarrays can be performed only in limited facilities, they also assessed miR expression by quantitative RT-PCR. Among several miRs with dysregulated expression, they identified miR-155 (increased in CTCL), miR-203 (decreased in CTCL), and miR-205 (decreased in CTCL) as the most discriminative set of miRs. Based on their expression levels, CTCL could be differentiated from BIDs with 91% sensitivity and 97% specificity and all MF cases irrelevant to their stages were accurately diagnosed. Afterwards, the result was validated using the other cohorts [51]. Moreover, Ralfkier et al. focused on the different miR profiles between early MF and AD and found 38 differentially expressed miRs [52]. Similar to the previous report, miR-155 was upregulated and miR-203 and miR-205 were downregulated in early MF compared to AD. Recently, plasma miR-155, miR-203, and miR-205 were also reported to be potential diagnostic tools for the diagnosis of MF and Sézary syndrome (SS) [53]. In 2018, Shen et al. proposed the other miR sets to distinguish CTCL including various subtypes from BIDs [54]. The sets included miR-155 (increased in CTCL), miR-200b (decreased in CTCL), miR-203 (decreased in CTCL), miR-142-3p (increased in CTCL), and miR-130b (increased in CTCL) and the classifier achieved 96% sensitivity and 72% specificity in the diagnosis of CTCL. However, based on their data, in early MF cases, miR-200b expression was not decreased and miR-130b expression was not increased. Thus, there may be a more suitable classifier for the differential diagnosis between early MF and BIDs. Collectively, miR analysis may help the diagnosis of early MF and can be widely used in the future, although quantitative RT-PCR cannot be performed in daily clinical practice in most facilities currently and a more suitable criteria for early MF diagnosis may be needed.

## 7. Conclusions

In this article, general methods and novel tools for diagnosis of early MF were summarized. The current diagnostic algorithm shows high sensitivity and specificity to some extent. However, there are still many cases difficult to distinguish between early MF and BIDs in daily clinical practice. In such cases, the detection of some molecules including TOX and CADM1, clonality analysis by NGS, and examination of miR expression might contribute to the diagnosis. There has been gradual increase in transcriptomic studies of MF [55]. Although skin samples of MF used in transcriptomic studies include many non-tumor cells, the exploration of the genome-wide expression of individual genes in skin samples may be useful in elucidating the pathogenesis and improving the diagnosis of MF. Litvinov et al. determined 17 gene sets that can distinguish MF and SS from BIDs [56]. The criteria have not been established yet, while such differentially expressed genes between early MF and BIDs may also help the diagnosis of early MF in the future. Having said that, those analysis cannot be usually conducted in many clinical facilities. Thus, repeated skin biopsy and gene analysis will be needed for the diagnosis of early MF in some cases. The most important point is that inappropriate systemic drugs, such as immunosuppressants and dupilumab, should not be started in cases suspected of CTCL. The establishment of more accurate and easier diagnostic methods and the dissemination of novel technologies are required to improve the management of patients suspected of early MF. 

## Figures and Tables

**Table 1:** Algorithm for diagnosis of early mycosis fungoides by Pimpinelli N et al. [17].

Criteria	Scoring System
Clinical Basic Persistent and/or progressive patches/thin plaques Additional (1) Non-sun-exposed location (2) Size/shape variation (3) Poikiloderma	2 points for basic criteria and 2 additional criteria 1 point for basic criteria and 1 additional criterion
Histopathological Basic Superficial lymphoid infiltrate Additional (1) Epidermotropism without spongiosis (2) Lymphocytic atypia	2 points for basic criteria and 2 additional criteria 1 point for basic criteria and 1 additional criterion
Molecular biology (1) Clonal T-cell receptor rearrangement	1 point for clonality
Immunopathological (Immunohistochemical) (1) <50% CD2+, CD3+, and/or CD5+ T cells (2) <10% CD7+ T cells (3) Epidermal/dermal discordance of CD2, CD3, CD5, or CD7 (T-cell antigen deficiency confined to the epidermis)	1 point for one or more criteria
